# Understanding what matters to metastatic castration‐resistant prostate cancer (mCRPC) patients when considering treatment options: A US patient preference survey

**DOI:** 10.1002/cam4.5313

**Published:** 2022-10-13

**Authors:** Daniel J. George, Ateesha F. Mohamed, Jui‐Hua Tsai, Milad Karimi, Ning Ning, Sayeli Jayade, Marc Botteman

**Affiliations:** ^1^ Duke University School of Medicine Durham North Carolina USA; ^2^ Bayer U.S. LLC Whippany New Jersey USA; ^3^ Evidence and Access OPEN Health Parsippany New Jersey USA; ^4^ Evidence and Access OPEN Health Rotterdam The Netherlands

**Keywords:** bone pain, metastasis, preferences, prostate cancer

## Abstract

**Background:**

Understanding how patients perceive the efficacy, safety, and administrative burden of treatments for metastatic castration‐resistant prostate cancer (mCRPC) can facilitate shared‐decision making for optimal management. This study sought to elicit patient preferences for mCRPC treatments in the US.

**Methods:**

We conducted a cross‐sectional survey using the discrete‐choice experiment method. Participants were asked to state their choices over successive sets of treatment alternatives, defined by varying levels of treatment attributes: overall survival (OS), months until patients develop a fracture or bone metastasis, likelihood of requiring radiation to control bone pain, fatigue, nausea, and administration (i.e., oral/IV injection/IV infusion). Using mixed logit models, we determined the value (i.e., preference weights) that respondents placed on each attribute. Relative attribute importance (RAI) and marginal rates of substitution (MRS) were calculated to understand patients' willingness to make tradeoffs among different attributes.

**Results:**

The final data set numbered 160 participants, with a mean age of 71.6 years old and a mean of 8.96 years since prostate cancer diagnosis. Participants' treatment preferences were as follows: OS (RAI: 31%), bone pain control (23%), nausea (16%), delaying fracture or bone metastasis (15%), fatigue (11%), and administration (3%). The MRS demonstrated that respondents were willing to trade 1.9 months of OS to eliminate moderate nausea and 3.3 months of OS for a reduction in fatigue from severe to mild.

**Conclusions:**

Improving OS is the highest priority for patients with mCRPC, but they are willing to trade some survival to reduce the risk of requiring radiation to control bone pain, delay a fracture or bone metastasis, and experience less severe nausea and fatigue.

## Graphical abstract

Understanding how patients perceive the efficacy, safety, and administrative burden of treatments for metastatic castration‐resistant prostate cancer (mCRPC) can facilitate shared‐decision making for optimal management. Our study shows that improving OS is the highest priority for patients with mCRPC, but they are willing to trade some survival to reduce the risk of requiring radiation to control bone pain, delay a fracture or bone metastasis, and experience less severe nausea and fatigue.

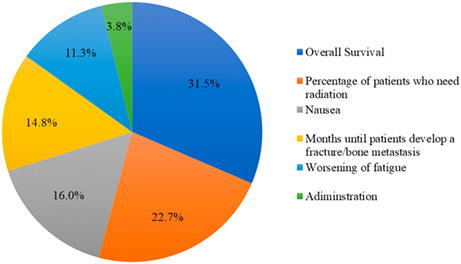

## 1 BACKGROUND

Prostate cancer (PC) is the most commonly diagnosed cancer and the second leading cause of cancer deaths for men in the United States (US).^1^ The cost of care for PC patients is estimated at $15,613 per patient per year,^2^ with a total of $16 billion across all PC patients per year in the US.^3^ Although most disease states of PC demonstrate relatively slow clinical progression over years, metastatic castration‐resistant prostate cancer (mCRPC) can have an aggressive disease course with clinical progression over months that leads to most PC‐related cancer deaths.^4^ CRPC can be defined as “disease progression despite androgen deprivation therapy (ADT) and may present as either a continuous rise in serum prostate‐specific antigen (PSA) levels, the progression of pre‐existing disease, and/or the appearance of new metastases.”^5^ mCRPC patients represent a subgroup of the CRPC patients in which there is evidence of metastasis.^6^, ^7^ Bone is the most frequently documented location of metastasis, accounting for over 70% of the occurrence of metastasis in mCRPC patients.^8^ Bone metastasis, skeletal‐related events, and pain are associated with higher mortality rates in mCRPC patients.^9^, ^10^

Various treatment options are available for the management of mCRPC, including immunotherapy (e.g., sipuleucel‐T), androgen receptor inhibitors (ARIs) (e.g., abiraterone acetate and enzalutamide), radioactive isotope agents (e.g., radium‐223 dichloride), chemotherapy agents (e.g., docetaxel, cabazitaxel, carboplatin), and in patients with homologous DNA repair defects, PARP inhibitors (olaparib and rucaparib) and platinum‐based chemotherapy.^4^, ^7^, ^11^, ^12^, ^13^ Other supportive therapies for mCRPC include bone‐health modifiers (e.g., denosumab, zolendronic acid) and external‐beam radiation therapy (EBRT).^11^ Because there are no curative treatments for mCRPC presently, the treatment goal should be to “provide the best possible quality of life (QOL) for as long as possible,” according to the American Society of Clinical Oncology (ASCO) and Cancer Care Ontario (CCO).^14^

The American Urological Association guidelines for CRPC states that “incorporating patients' preferences and personal goals should be implemented when choosing management strategies.”^15^ Further, research has shown that patients and urologists differ in how they make trade‐offs between different characteristics of prostate cancer treatments.^16^ Given that treatment options for mCRPC vary in benefits, risk profiles, and modes of administration, understanding patients' perspectives on how they value individual treatment characteristics can offer clinicians critical insights.

Incorporating patient preferences into clinical practice has been shown to improve health outcomes and patients' satisfaction of care.^17^, ^18^ In a randomized controlled trial of 743 PC patients, clinicians discussed treatment options after receiving treatment preference results expressed by the patients. Patients with PC demonstrated their treatment preferences through importance‐ranking exercises in which they chose between hypothetical treatment options. Compared with normal care, the intervention group had statistically significant (*p* < 0.05) fewer severe urologic symptoms, a lower likelihood of having a high depression score, less decline in physical and social functioning scores, and a greater satisfaction with care.^17^, ^18^

A few studies have been conducted in Europe and Asia to understand mCRPC or CRPC patients' preferences for prostate cancer treatment options.^19^, ^20^ Eliasson et al. demonstrated that mCRPC patients in France, Germany, and the UK have a strong preference for treatments that can fully control bone pain.^19^ Uemura et al. showed that fatigue and a reduction in bone pain are the most important treatment features for CRPC patients studied in Japan.^20^ However, no study has examined the value that US patients place on mCRPC treatments' different attributes. The goal of this study was to quantify treatment preferences of mCRPC patients and to understand which key treatment attributes (e.g., efficacy, adverse events [AEs], administration) have the most impact in driving the treatment decisions of mCRPC patients in the US.

## 2 MATERIAL AND METHODS

### 2.1 Study design and sample

A cross‐sectional survey was conducted using the discrete‐choice experiment (DCE) method. Participants were asked to state their choices over a successive sets of treatment options, defined by varying levels of treatment characteristics (i.e., attributes). DCE is a rigorous research method that has been used by researchers, health economists, and regulatory bodies to quantify patient preferences.^21^, ^22^

The study comprised a qualitative and a quantitative phase. The qualitative phase involved a targeted literature review and qualitative interviews with clinicians and patients to identify and subsequently refine our understanding of what treatment attributes matter to patients in their treatment choices. This phase also included an assessment of the appropriate “levels” (i.e., amount of intensity) each attribute should take to have a potential impact on treatment choice. The quantitative phase consisted of activities of drafting the DCE survey, pretesting the survey with patients, programming the survey, recruiting survey participants, and analyzing the data.

Clinicians experienced with treating mCRPC (participated in qualitative phase) and mCRPC patients (participated in qualitative and quantitative phases) were recruited by Global Perspectives (GP) through their online healthcare provider database and patient panels. Patients with mCRPC were eligible for the study if they were 18 years or older, self‐reported a clinician‐confirmed diagnosis of mCRPC, resident of the United States, able to read and speak English, and able to provide informed consent. Quota sampling was used in patient recruitment to ensure that the study sample was representative of the US prostate cancer population by age. During the screening process, patients were screened out once the age quota for each group is fulfilled. This allowed the sample to be generally consistent with the age distribution data from the National Cancer Institute Surveillance, Epidemiology, and End Results program.^23^ Clinicians were eligible if they were urologists/oncologists, were 18 years or older, provided treatment to ≥5 mCRPC patients a month, treated patients in a hospital‐based or freestanding clinical practice located in the United States or Canada, and able to read and speak English and provide informed consent.

The study was designed and conducted following good research practices for conjoint analysis, including DCEs, and statistical methods recommended by the International Society for Pharmacoeconomics and Outcomes Research (ISPOR) Conjoint Analysis Good Research Practices Task Force.^24^, ^25^ It was deemed exempt from further institutional review board (IRB) review by the Advarra IRB, a centralized IRB in the United States (US) (Protocol number: 21157). Written informed consent was acquired for physician interviews. A waiver of written consent for patient interview was obtained through Advarra IRB's review and patients provided verbal consent on recording at the beginning of the interviews. Patients who participated in the DCE survey provided online consent prior to initiating the survey.

### 2.2 Attribute and levels of development

A total of 6 treatment attributes were examined in this DCE, including efficacy attributes (i.e., overall survival [OS], months until patients develop a fracture or bone metastasis, likelihood of requiring radiation to control bone pain), risk attributes (i.e., fatigue and nausea), and administration attributes (i.e., mode of administration). The levels ascribed to each of these attributes were selected to reflect clinical practice, and the description of the levels was constructed by adapting the definitions of Common Terminology Criteria for Adverse Events (CTCAE) v5.0^26^ to patient‐friendly language, where mild, moderate, and severe corresponded to Grade 1, 2, and 3 AEs, respectively. The final attributes and levels included in the DCE are presented in Table 1.

**TABLE 1:** Attributes and levels included in the discrete‐choice experiment

Attributes	Levels
Overall survival	12 months 16 months 22 months
Months until patients develop a fracture or bone metastasis	10 months 14 months 20 months
Percentage of patients who need radiation to control bone pain	20% of patients will need radiation for severe bone pain 30% of patients will need radiation for severe bone pain 50% of patients will need radiation for severe bone pain
Worsening of fatigue	Remain at mild, no worsening Worsening from mild to moderate Worsening from mild to severe
Nausea	None Mild Moderate Severe
Administration	Daily oral pills (one or more times daily) IV injections (1‐min, every 3 weeks) IV infusions (1‐hour, every 4 weeks)

A rigorous process was used to identify the attributes and corresponding levels included in the study. First, the study team conducted a targeted literature review of clinical trials of relevant mCRPC treatments (e.g., trials including enzalutamide, RA‐223, and docetaxel as comparators)^27^, ^28^, ^29^ and previous preference studies^19^, ^20^ conducted in this space. A list of preliminary attributes and corresponding levels was generated based on the literature review, and the list was presented to six clinicians during one‐on‐one qualitative interviews to elicit feedback on the relevance of the attributes in mCRPC. Clinicians confirmed the relevance of the attributes through rankings and interview discussions. Overall, they valued efficacy over risk attributes with OS named the most important attribute across the board. Bone pain and time to symptomatic skeletal event (SSE) were also noted as important efficacy attributes. For example, one clinician noted, *“I perceive that (bone pain) as one of the most troublesome symptoms from metastatic disease.”* Based on the interviews with clinicians and discussion in the research team, we refined the attribute list to six items, namely OS, time to SSE, reduction in opioid‐managed bone pain, fatigue, nausea, and administration. This refined attribute list and attribute levels were subsequently used for the construction of an experimental design consisting of the statistical arrangement of the attributes and levels in the survey and the DCE survey itself. The attributes and levels were further revised and refined after survey pretest, pilot, and soft launch.

### 2.3 Survey design and development

A draft survey was developed after the qualitative interviews. The study team then conducted pretest interviews with 10 patients, a pilot study with 10 patients, and a soft launch with 30 patients.

During the pretest interviews, mCRPC patients used the “think‐aloud” technique to explain their thinking process while completing the survey.^30^ Patients confirmed the refined attribute list encompassed all the important aspects of their treatment decision‐making. They were also provided the opportunity to raise any missing but relevant attributes; however, no singular attribute was mentioned by more than one patient. Based on the pretest findings, the study team revised the attributes, levels, and survey questions. For example, nearly all pretest patients were challenged by the acronym and concept of SSE. Thus, the attribute was revised from “time to SSE” to “months before you develop a fracture or painful bone metastasis that requires surgery or radiation.”

During the pilot and the soft launch, patients answered the online survey, and the study team analyzed their responses to the comprehension‐check questions. Additionally, the study team used a multinomial logit (MNL) model to analyze the choice tasks of the soft‐launch dataset. Based on the pilot and soft‐launch results, the study team further revised the attributes, levels, attribute explanations, and the survey design as well as added examples and a tutorial video to clarify challenging concepts for the patients and to improve survey clarity.

**FIGURE 1:**
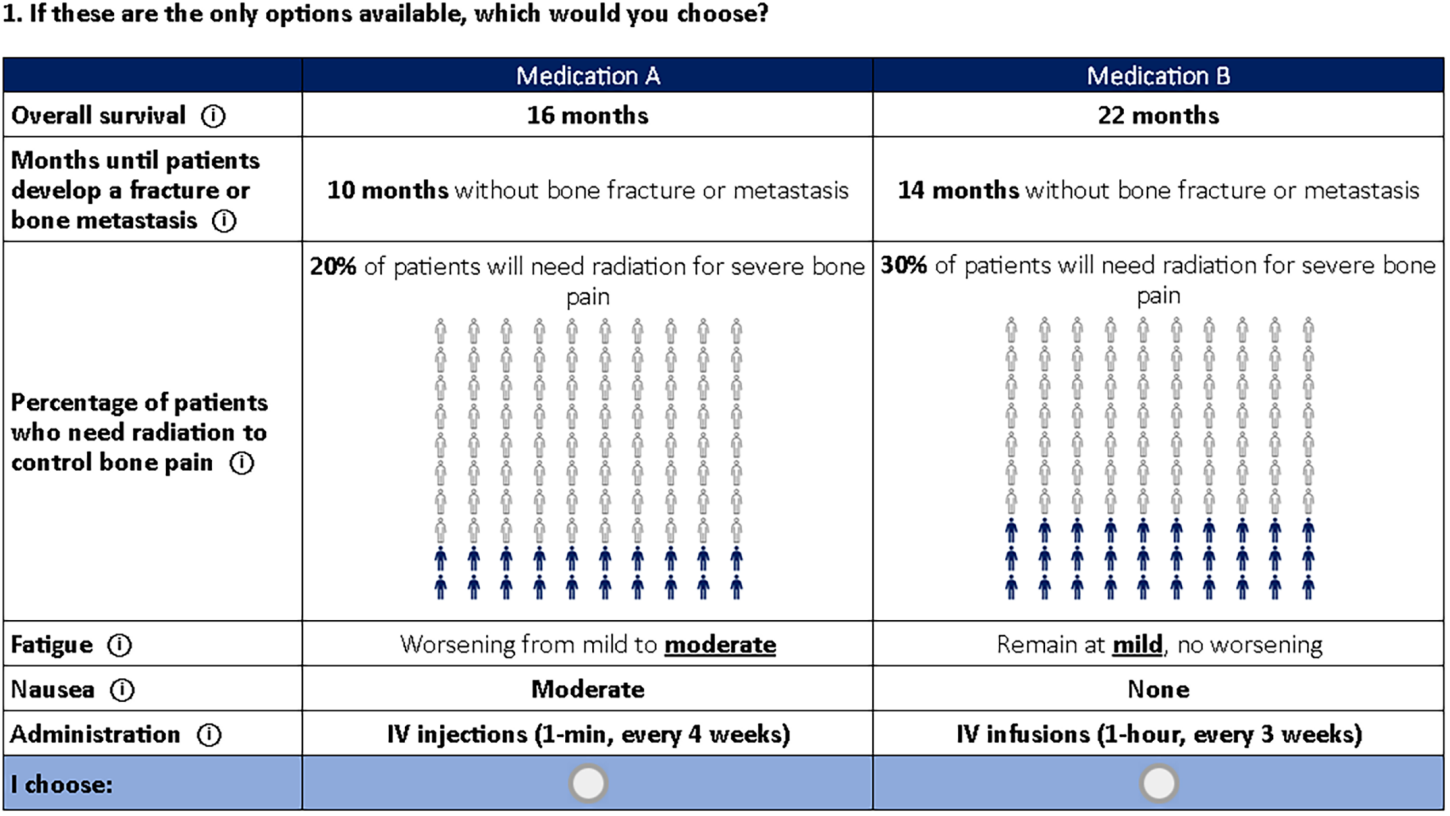
Example of choice question in the discrete‐choice experiment

The final study survey included five sections: sociodemographic characteristics, disease and treatment experience, attribute descriptions and comprehension checks, and the DCE choice tasks. An example of a choice task included in the DCE survey is shown in Figure 1. Brief descriptions, visual aids, examples, and a tutorial video were used to introduce the attributes and levels to the participants. Comprehension‐check questions were simple exercises focusing exclusively on one attribute at a time to assess respondents' comprehension of each attribute.

The DCE choice task is determined by the experimental design, which dictates the number of choice sets, the number of alternatives per choice set, the number of attributes, and the combination of the attribute levels in each choice set.^31^ For this study, we constructed a D‐efficient design using SAS to generate a series of profiles that were statistically efficient (i.e., minimized the variances and covariances of the parameter estimates).^32^ The design consisted of 52 choice sets blocked into 4 versions of 13 choice sets each, where each respondent was randomly assigned to one of the four blocks to avoid respondent fatigue. Choice sets were restricted to those where OS was equal or longer than bone pain control to avoid illogical combinations. Details on the D‐efficient design and additional quality checks are included in Appendix A and Appendix B, respectively.

### 2.4 Statistical analysis

Data analyses for the DCE survey were conducted using Stata® 14.2 and SAS/STAT® 14.3. Descriptive statistics were used to analyze responses for the sociodemographic and clinical characteristics as well as the comprehension check questions. Additionally, the study team searched for any explanatory quotes in the free text section. Effects‐coded random parameters logit (RPL) models (i.e., mixed logit models) were used to examine the choice responses^24^ in the form of preference weights estimation. Preference weights signify the relative value respondents place on each attribute/level, where a higher weight indicates a stronger preference.

The relative attribute importance (RAI) scores were computed to understand the importance patients place on each of the attributes and the degree of sensitivity to which patients respond to the change of the levels within each attribute. RAI scores are calculated by (i) summing the differences in preference weights of each attribute's best (highest preference weight) and worst (lowest preference weight) levels across all attributes, then (ii) dividing the differences in preference weight of the best and worst levels of each attribute by the overall variations in preference weight across all attributes. A higher RAI score indicates both that the attribute has a greater impact on how patients make their decisions and that patients are more sensitive to the change in levels, but only in the context of the levels presented in the survey. Further details on the RPL model and RAI score calculations are included in Appendix B.

The marginal rates of substitutions (MRSs) were calculated to understand the degree to which patients were willing to trade off between each attribute. The log‐likelihood and model fit statistics were used to optimize the model, which demonstrated that it was more appropriate to categorize the bone pain attribute with a linear parametrization (Log‐likelihood: −1019.00, Akaike Information Criterion [AIC]: 2086.01) compared to a categorical parametrization (Log‐likelihood: ‐1021.15, AIC: 2094.29). Thus, bone pain was specified as a linear variable in the MRS analysis. Through the MRS analysis, we estimated the amount of OS that patients would be willing to forego for various improvements in the levels of the other five attributes.

## 3 RESULTS

### 3.1 Sample characteristics

**FIGURE A1:**
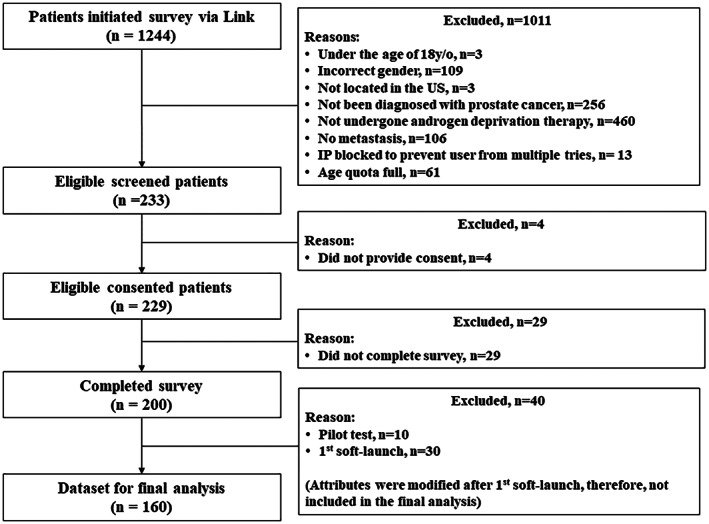
Flowchart of patient survey respondent selection. US, United States.

Of the 1244 respondents who accessed the online survey, 233 (18.7%) patients were eligible. Among the eligible patients, four did not provide consent to participate and another 29 did not complete the survey. Of the remaining 200 respondents, 40 patients who participated in the pilot test or soft launch were excluded from the analysis since the attributes and levels were modified after the soft launch and varied from those used in the final sample, which contained a total of 160 completed responses. A flowchart detailing the respondent selection is presented in Figure A1.

The sociodemographic and clinical characteristics of the patients are summarized in Table 2. The mean age of the respondents was 71.61 years old (standard deviation [SD]: ±10.74). Most of the patients were white (69.4%), married (72.5%), had a college education or university degree (37.5%), lived in urban area (54.4%), and were retired (62.5%). The average duration from the diagnosis of prostate cancer was 8.96 (±6.08) years. The most reported cancer metastasis location was lymph nodes (56.3%), followed by bone (41.3%) and lung (20.0%). Most frequently reported current medication use was docetaxel (26.9%), followed by bicalutamide (17.5%) and leuprolide (15.0%).

**TABLE 2:** Sociodemographic characteristics of patients (*N* = 160)

Characteristic	Category	Statistics
Age in years, Mean (SD)		71.61 (10.74)
Gender, *n* (%)	Male	160 (100.0%)
Race, *n* (%)	White	111 (69.4%)
	Black or African American	25 (15.6%)
	Asian	12 (7.5%)
	Middle Eastern or North African	6 (3.8%)
	Native Hawaiian or other Pacific Islander	4 (2.5%)
	Other	2 (1.3%)
Urban/rural, *n* (%)	Urban	87 (54.4%)
	Suburban	60 (37.5%)
	Rural	13 (8.1%)
Marital status, *n* (%)	Single, never married	5 (3.1%)
	Married or domestic partner	116 (72.5%)
	Divorced	16 (10.0%)
	Separated	4 (2.5%)
	Widowed	19 (11.9%)
Education, *n* (%)	Some college or certification program	22 (13.8%)
	College or university degree (2‐ or 4‐year)	60 (37.5%)
	Advanced or graduate/post‐graduate degree	47 (29.4%)
	Other	1 (0.6%)
Employment, *n* (%)	Employed full‐time	41 (25.6%)
	Employed part‐time	6 (3.8%)
	Retired	100 (62.5%)
	Unemployed	3 (1.9%)
	Disabled	10 (6.3%)
Time from diagnosis of prostate cancer in years, Mean (SD)		8.96 (6.08)
Location where cancer spread, *n* (%)^a^	Spread to bone	66 (41.3%)
	Spread to liver	8 (5.0%)
	Spread to lung	32 (20.0%)
	Spread to lymph nodes	90 (56.3%)
	Spread to other	7 (4.4%)
	Not sure	3 (1.9%)
Current medication, *n* (%)	Docetaxel (Taxotere®)	43 (26.9%)
	Cabazitaxel (Jevtana®)	8 (5.0%)
	RA‐223 (Xofigo®)	3 (1.9%)
	Sipuleucel‐T (Provenge®)	6 (3.8%)
	Enzalutamide (Xtandi®)	11 (6.9%)
	Abiraterone (Zytiga®)	9 (5.6%)
	Goserelin (Zoladex®)	22 (13.8%)
	Bicalutamide (Casodex®)	28 (17.5%)
	Triptorelin (Trelstar®)	4 (2.5%)
	Leuprolide (Eligard®/Lupron®)	24 (15.0%)
	Other	2 (1.3%)
Developed a fracture or bone metastasis		79 (49%)
Experienced severe bone pain that required radiation		62 (39%)

*Note*: Due to rounding, percentages may not add up to 100%.

^a^ Participants were able to select multiple options regarding the location where cancer has spread.

The result of the comprehension test showed that patients understood the survey contents well, with 91% and 98% of patients passing the comprehension test for “months until patients develop a fracture or bone metastasis” and “percentage of patients who need radiation to control the bone pain,” respectively. Quality checks were conducted and confirmed that the full data set can be used. Details of the quality checks are included in Appendix A.

### 3.2 Patient treatment preferences and relative importance of attributes

**FIGURE 2:**
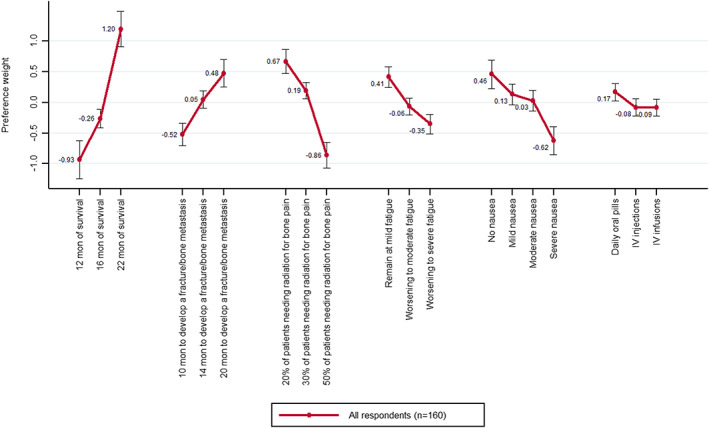
Patient treatment preferences estimated in preference weights. IV, intravenous; mon, month. Vertical bars denote the 95% confidence intervals around estimates.

Patients' treatment preferences estimated in preference weights and their corresponding 95% confidence intervals (CIs) are presented in Figure 2 and Annex Table B1. The estimated preference weights are only meaningful when compared to each other, i.e., the relative differences matter. In this study, they were ordered rationally, where better levels for each attribute were preferred (i.e., better efficacy or fewer side effects) to worse levels. The difference in preference weights of adjacent levels indicates the relative impact of shifting from one level to the next, where a larger difference means a greater impact on treatment choices. For example, worsening from mild fatigue to moderate fatigue (0.47) is about the same as the change in preference weights from 20% of patients to 30% of patients needing radiation to control bone pain (0.48).

**FIGURE 3:**
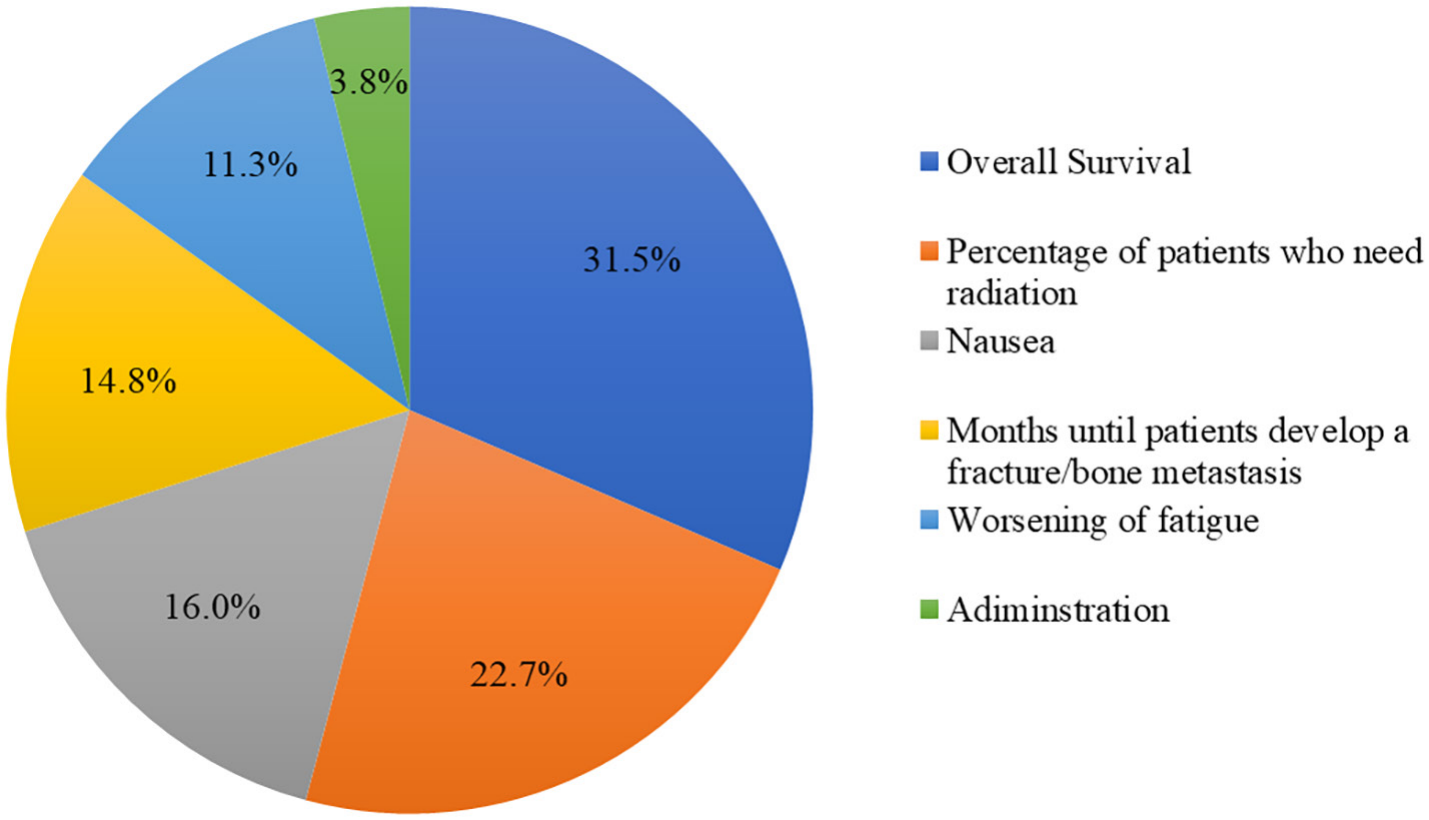
Relative importance scores of treatment attributes

To understand the importance that patients place on each of the attributes, RAI scores were calculated; they are presented as a pie chart in Figure 3. RAI scores demonstrated patients' preferences for mCRPC treatments characteristics as followed (in decreasing order of importance over the attribute ranges included in the study): OS (RAI score: 31.5%), reduction in the need for bone pain control (22.7%), nausea (16.0%), months until patients develop a fracture or bone metastasis (14.8%), fatigue (11.3%), and mode of administration (3.8%).

### 3.3 Trade‐offs between overall survival and other attributes

**FIGURE 4:**
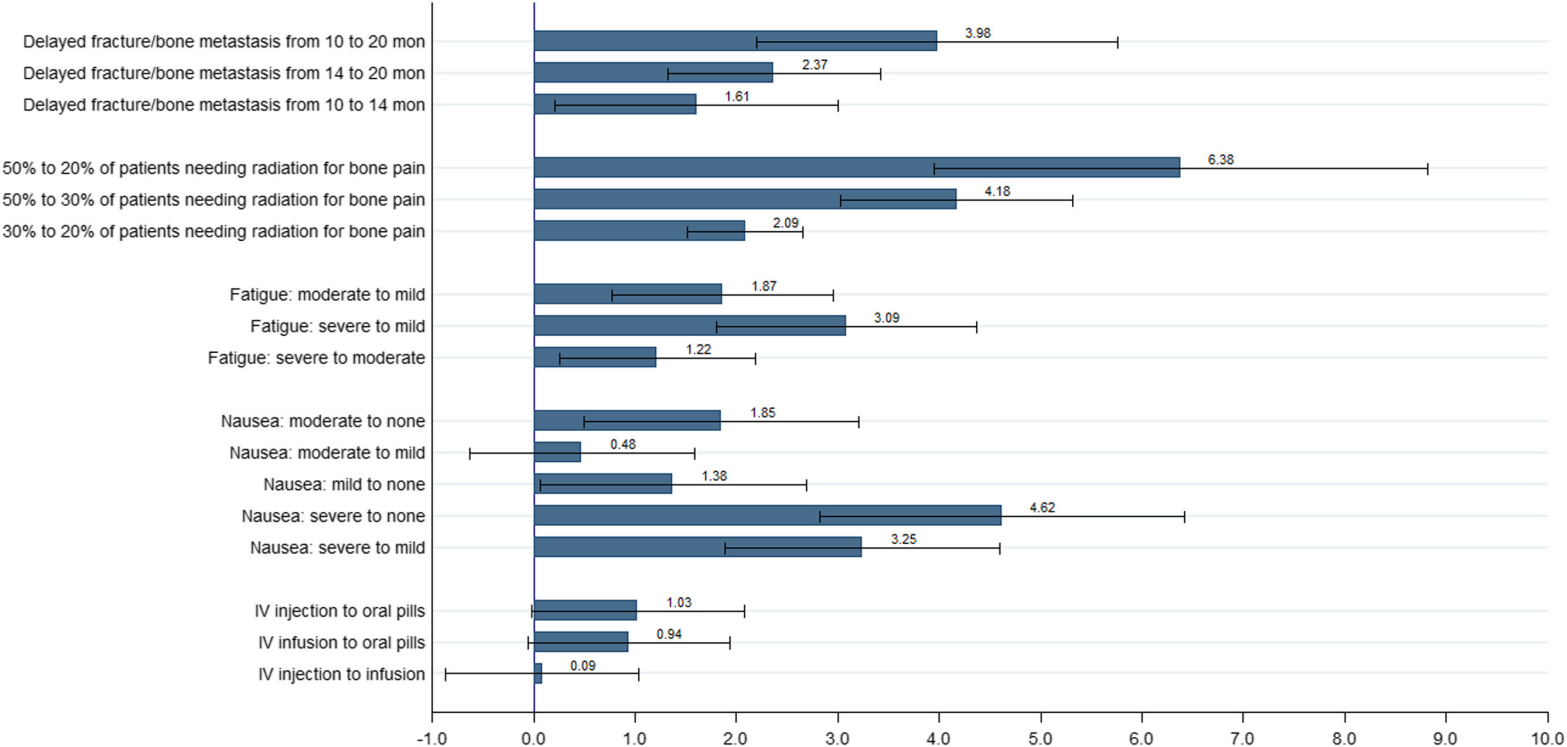
Reduction in months of overall survival estimates. IV, intravenous; mon, months.

The amount of OS that patients were willing to forego for various improvements in the other 5 attributes and their corresponding 95% CIs are presented in Figure 4. We found that patients were willing to trade more months of OS for improvement in treatment benefits and a reduction in the risks that they consider important. For example, patients were willing to trade 3.98 months of survival for a 10‐month improvement in duration before developing a fracture or painful bone metastasis. Patients were also willing to trade 6.38 months of OS for a reduction in risk (50% to 20%) of requiring radiation for controlling bone pain. To reduce nausea and fatigue from severe to none, patients were willing trade 3.09 months and 4.62 months of OS, respectively. Patients were willing to trade slightly more OS time to reduce their risks in fatigue from moderate to mild than from severe to moderate (1.87 vs. 1.22 months, respectively). In contrast, patients did not have a strong willingness to trade OS for a reduction in nausea from moderate to mild or any changes between modes of administrations (not statistically significantly different from zero).

The results were heterogeneous, with a subset of patients unwilling to compromise any trade‐off of their top priority. Out of the 160 mCRPC patients, 27 (16.9%) dominated on one single attribute and did not make trade‐off behavior. Attribute dominance was most observed with OS, where 18 patients (11.25%) dominated on OS only. Fewer than 2% of patients showed an attribute dominance on the other five attributes (months before developing a fracture/painful bone metastasis, 1.3%; bone pain, 0.6%; fatigue, 0.63%; nausea, 1.9%; administration, 1.3%).

## 4 DISCUSSION

Patient preference studies are useful to clinicians in order to better understand the patient population they are treating and how patient preferences vary across that population. In this context, there are few studies that have systematically evaluated preferences in patients with mCRPC. Our study has several key findings. First, among the range of efficacy, risks, and administration examined in the study, mCRPC patients on average prioritized efficacy over side effects over administration. This is also evident from the 18 patients (11.25%) who dominated on OS, meaning that they always choose the treatment that offers the highest OS regardless of other attributes. These results reinforce the value of treatments that prolong OS and the need to develop further therapy for this patient population. That said, most patients were willing to trade some OS with other risk attributes, demonstrating that maintaining QOL is important to mCRPC patients even when compared to OS. This makes sense when we consider the incurable status of these patients. With limited life expectancy, many patients will put increasing importance on the quality of survival.

In terms of what is most important to patients, OS and bone pain control were the most important efficacy attributes, and nausea was the most important risk attribute. Patients were willing to trade more months of OS for a reduction in risks or for an improvement in efficacy that they consider more important, particularly a reduction in risk of patients requiring radiation for bone pain from 50% to 20% (worth 6.38 months of OS) and an improvement of nausea from severe to none (worth 4.62 months of OS).

Bone pain control remains an important issue in PC. In our study, we found that mCRPC patients consider bone pain as the most important attribute besides OS. This is consistent with findings from other preference studies in PC. One study showed that patients with metastatic hormone‐sensitive prostate cancer (mHSPC) in Europe considered bone pain control the second most important attribute, just behind overall efficacy of delaying disease progression.^33^ Another study demonstrated that CRPC patients in Japan considered a reduction in bone pain control the most important efficacy attribute, even more so than OS.^20^ Yet another study showed that mCRPC patients in Europe considered bone pain control to be most important and were 12‐fold more likely to prefer treatments associated with no bone pain than treatments associated with uncontrolled bone pain (odds ratio [OR] = 12.07 [95% CI,10.56–13.80]).^19^

Nausea and fatigue are important side effect aspects related to mCRPC treatments that were included in our survey. One prior preference study that surveyed mHSPC patients found that nausea was the most important side effect for patients.^33^ In another preference study, CRPC patients considered fatigue as the most important attribute, surpassing OS.^20^ Fatigue was also noted by advanced PC patients as one of the most frequently occurring and most severe symptoms.^34^ Of all of the AE levels presented, mCRPC patients were seen to avoid treatments associated with severe nausea the most (preference weight − 0.62), defined as nausea requiring tube feeding/total parenteral nutrition (TPN)/hospitalization, followed by the avoidance of severe fatigue (preference weight − 0.35), defined as fatigue limiting self‐care. Additionally, when comparing the different levels within each of the AEs, we found that the key drivers of patients' decisions (indicated by the biggest change in preference weights) were avoidance of going from moderate to severe nausea (preference weight change of 0.65) and avoidance of a progression from mild fatigue to moderate fatigue (preference weight change of 0.47).

Studies have found that both physicians and patients prefer a collaborative decision‐making process when it comes to treatment decisions. A study of 1050 clinicians showed that the majority (75%) of clinicians preferred to participate in shared‐decision making (SDM) with their patients, while 14% preferred paternalism and 11% preferred consumerism.^35^ Another study surveying 7169 PC patients showed that collaborative decision‐making (62.2%) and patients taking on an active role (32.4%) were most preferred, with only 5.5% of patients preferring a passive role when it comes to their treatment decision‐making.^36^ The American Urological Association also considers SDM to be critical in the delivery of high‐quality care in urological practices and relevant to reimbursement models in the future.^37^ The findings of this study offer clinicians key insights on what mCRPC patients consider important treatment characteristics, which can in turn aid in the SDM process. Additionally, clinicians should fairly discuss both efficacy and side effects when presenting treatment options to mCRPC patients, as our study has shown that both aspects are important to patients' treatment decision‐making.

There are a few strengths to this study. To the best of our knowledge, it is the first to examine treatment preferences of mCRPC patients in the US. Additionally, we used age quota sampling to reach a sample that is representative of the prostate cancer population in the US. This approach ensured that the perspectives of older prostate cancer patients were represented in the study, whereas in other studies, patients recruited through online panels often skewed younger.

The median age of our study sample was 72 years old, with 76.8% of the patients 65 years old or older, which is comparable with a published claims database study of 3690 mCRPC patients in the US, where the median age was 72 years old and 72% of patients were on Medicare (i.e., 65 years old or older).^38^ With a mean age of 71.6 years old, our sample is also older than the mean age of other preference studies for prostate cancer in Western countries, which ranges from 64.2 to 70.7 years old.^19^, ^39^

This study is subject to some limitations. First, DCEs are subject to hypothetical bias since respondents were providing their input on hypothetical profiles.^40^ To attenuate hypothetical bias, rigorous qualitative work via pre‐testing was undertaken to ensure that the treatment profiles were meaningful to patients and that attributes and levels included made sense to them.

Second, the study findings are restricted to the attributes and levels presented in the survey. By going through a rigorous attribute and level selection process and soliciting both clinician and patient input, we believe that the attributes presented are critical to the mCRPC population, although one should not extrapolate beyond the attributes/levels included in the survey when interpreting the study results.

Third, though the study captured the time since diagnosis and whether patients had experienced certain symptom effects such as fracture, bone metastasis, and severe bone pain, the study did not investigate whether patients' preferences differed by their time course along mCRPC and previous experiences as the aim of the study was to investigate the average preferences among the mCRPC patient population.

Fourth, the study team did not specify in the description of the risk attributes (i.e., fatigue and nausea) how long these adverse events (AEs) will last. It is possible that patients may have different assumptions on how long the AEs will last and that these assumptions may have shaped how they answered the choice task. However, the study team did not note any particular struggles or inquiries from the patients during the survey pretest interviews on the duration of the AEs. Additionally, a number of past preference studies surveying the prostate cancer patient population have included AEs, an none of which have specified the duration of the AEs.^19^, ^20^, ^33^, ^41^ In particular, Srinivas et al., de Freitas et al., Eliasson et al., and Uemura et al. have all included fatigue as an attribute,^19^, ^20^, ^33^, ^41^ and de Freitas et al. have included nausea as an attribute,^33^ all without specifying the estimated duration for when patients experienced fatigue or nausea.

Lastly, the use of online panels as the main source for recruitment may have limited the generalizability of the results to the wider mCRPC population. For example, 86% of the respondents completed a college or a higher educational degree, indicating that the participants are more educated than the general population but on par with another published patient preference study in CRPC (i.e., 84% college and above). Additionally, most of the participants in this study were Whites (69.4%), followed by Blacks or African Americans (15.6%). Compared to other online survey studies (4%–11.2%) in prostate cancer in the US,^41^, ^42^ our study has a higher representation of Black or African American participants. However, Black or African American men have the highest incidence rate and present with a more aggressive disease outlook in prostate cancer.^43^ The underrepresentation of Black or African American participants is a common challenge across web‐based survey studies, where Black or African American participants are found to be less likely to provide consent in web‐based survey studies and are less likely to complete the survey.^44^ We recognize that systemic racism has added to the underrepresentation of Black or African American men in studies. While our study did not adequately address this concern, the 15.6% representation of Black men is above the national representation of Black men in the US population (roughly 14%). More studies dedicated to underserved populations are needed.

## 5 CONCLUSIONS

Improving OS remains the highest priority, but mCRPC patients are willing to sacrifice some survival to reduce the risk of requiring radiation to control bone pain, delay a fracture or bone metastasis, and experience less severe nausea and fatigue.

## AUTHOR CONTRIBUTIONS

**Daniel J George:** Conceptualization (equal); supervision (equal); validation (equal); writing – original draft (equal); writing – review and editing (equal). **Ateesha Mohamed:** Conceptualization (equal); funding acquisition (equal); investigation (equal); methodology (equal); supervision (equal); writing – original draft (equal); writing – review and editing (equal). **Jui‐Hua Tsai:** Conceptualization (equal); formal analysis (equal); funding acquisition (equal); investigation (equal); methodology (equal); project administration (equal); supervision (equal); validation (equal); writing – original draft (equal); writing – review and editing (equal). **Milad Karimi:** Conceptualization (equal); formal analysis (equal); investigation (equal); methodology (equal); project administration (equal); supervision (equal); validation (equal); writing – original draft (equal); writing – review and editing (equal). **Ning Ning:** Formal analysis (equal); investigation (equal); methodology (equal); project administration (equal); writing – original draft (equal); writing – review and editing (equal). **Sayeli Jayade:** Conceptualization (equal); formal analysis (equal); project administration (equal); writing – original draft (equal); writing – review and editing (equal). **Marc Botteman:** Conceptualization (equal); formal analysis (equal); funding acquisition (equal); investigation (equal); methodology (equal); project administration (equal); supervision (equal); validation (equal); writing – original draft (equal); writing – review and editing (equal).

## FUNDING INFORMATION

This study received funding support from Bayer Pharmaceuticals.

## CONFLICT OF INTEREST

DG is a clinical advisor of Bayer Pharmaceuticals and received consulting fees from Bayer Pharmaceuticals for this study. AFM is an employee of Bayer Pharmaceuticals. JHT, MK, NN, SJ, and MB are employees of OPEN Health, a research and consulting company that received research funding from Bayer Pharmaceuticals for this study. In addition, MB owns shares of OPEN Health.

## ETHICS APPROVAL STATEMENT

This study was deemed exempt from further institutional review board (IRB) review by the Advarra IRB, a centralized IRB in the United States (US) (Protocol number:21157).

## PATIENT CONSENT STATEMENT

Written informed consent was acquired for physician interviews. A waiver of written consent for patient interview was obtained through Advarra IRB's review and patients provided verbal consent on recording at the beginning of the interviews. Patients who participated in the discrete‐choice experiment (DCE) survey provided online consent prior to initiating the survey.

## PERMISSION TO REPRODUCE MATERIAL FROM OTHER SOURCES

Not applicable.

## CLINICAL TRIAL REGISTRATION

Not applicable.

## Figures and Tables

**FIGURE B1:**
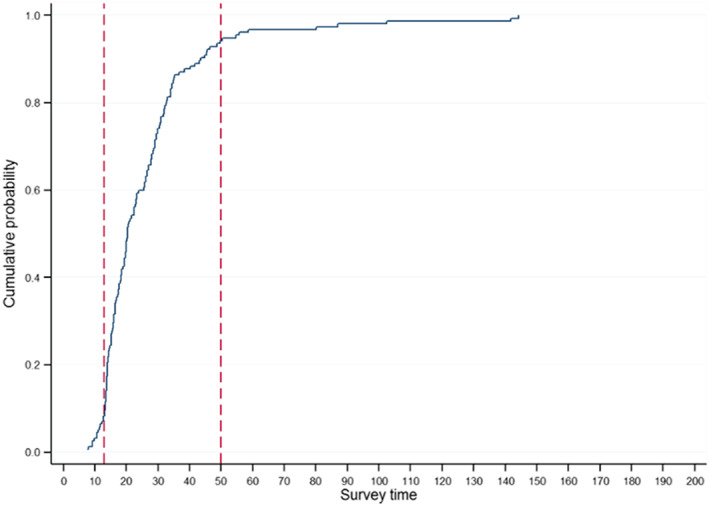
Time patients took to complete the survey

## Data Availability

The dataset of the DCE survey is available from the corresponding author upon reasonable request.

## APPENDIX A STATISTICAL METHODS (INCL. D‐EFFICIENT DESIGN, RPL MODELS, AND RAI SCORES)

### D‐efficient design

The DCE design is described by the number of choice sets, the number of alternatives per choice set, the number of attributes and the combination of the attribute levels in each choice set.^31^

A full factorial design—i.e., one in which all possible combinations of attributes and levels are included—was not feasible. Five attributes with 3 levels each and one attribute with 4 levels would result in a total of 471,906 (= 3^5^ × 4^1^× [(3^5^ × 4^1^) ‐ 1]/2) possible combinations of two‐alternative choice tasks. This number of combinations is too large and impractical to be implemented in a survey study. Hence, a D‐efficient design was used to extract a subset of profiles that were statistically efficient (i.e., minimized the variances and covariances of the parameter estimates).^32^

The DCE design was constructed in SAS using a commonly used algorithm developed by Kuhfeld et al and followed recommendations from the ISPOR Good Research Practices for Conjoint Analysis Task Force.^32^, ^45^ The final design consisted of 52 choice sets blocked into 4 versions of 13 choice sets each. The purpose of blocking the design into smaller subsets was to ensure that each respondent received a manageable number of choice sets to reduce respondent burden. Each patient received a random block version. Choice sets were restricted to those where OS was equal or longer than bone pain control to avoid illogical combinations.

### Random parameters logit (RPL) models

Random parameters logit (RPL) models (i.e., mixed logit models) were used to examine the choice responses. In the standard Random Utility Theory, a utility function for each decision maker *n* for each alternative j ($u_{\mathrm{nj}}$) consists of a fixed component ($v_{\mathrm{nj}}$) and an unobserved random component ($\varepsilon_{\mathrm{nj}}$):

$$u_{\mathrm{nj}}=v_{\mathrm{nj}}+\varepsilon_{\mathrm{nj}}$$

For this Discrete choice experiment, the deterministic component was defined as a linear function of the attributes shown to respondents at each task, t:

All attributes were effects‐coded, where the omitted level in each attribute is set to the negative sum of other levels. The $\varepsilon_{\mathrm{njt}}\;$ term is independent and identically distributed and extreme value random term. In addition, the random parameters logit (RPL) models assumed preference with each $\beta_{\mathrm{kn}}$ having an independent normal distribution with mean and standard deviation that were estimated.

### Relative attribute importance (RAI) scores

The vertical distance between an attribute's best and worst levels (i.e., difference between highest and lowest preference weights) is also a measure of the overall average relative importance of the attribute over the ranges represented in the DCE. Relative attribute importance (RAI) scores were also calculated by expressing this vertical distance as a percentage of the total variance observed (i.e., summation of vertical distances across all attributes):

$${\mathrm{RAI}}_{i}=\frac{\max\left(\beta_{i}\right)-\min\left(\beta_{i}\right)}{\sum_{n=1}^{n}\left(\max\left(\beta_{n}\right)-\min\left(\beta_{n}\right)\right)}*100$$

where given a range of attributes n, $\max\left(\beta_{i}\right)\;$ is the prefernece weight of the most desirable level of attribute i and $\min\left(\beta_{i}\right)$ the preference weight of the least desirable level of attribute i. The larger the RAI score, the more sensitive choices were to variation in the levels of a treatment attribute.

## APPENDIX B QUALITY CHECKS AND SENSITIVITY ANALYSIS

Quality checks were conducted to assess the data quality of the respondents' responses. In addition to the 13 main choice tasks, each participant was presented with two additional tasks (i.e., one repeat choice task and one dominant choice task) that served as quality checks. The repeat task was a repeat of one of the 13 tasks and was used to discern whether participants were consistent in their choice behavior. The dominant task presented the respondents with a profile that was superior in all the attribute levels (i.e., better efficacy and fewer side effects) to the other profile and was used to examine whether respondents were making rational choices.

Additionally, the study team also checked for task non‐attendance by identifying straight‐liners who always select the treatment option shown in the same position of the choice task (e.g., always chooses treatment A) regardless of the attribute/levels presented.^46^ Attribute dominance was also assessed, which examined the occurrence of when patients make their decisions by focusing solely on one singular attribute and were unwilling to make trade‐offs.^46^

Sensitivity analyses were conducted with the exclusion of respondents (i) who were straight‐liners and (ii) who failed either the dominance test or the repeated test and completed the survey in a very short (i.e., <13 min) or long (i.e., >50 min) amount of time. The cumulative probability of time patients took to complete the survey is presented in Figure B1. It demonstrates that most patients completed the survey between 13‐50 minutes and that the appearance of kinks in the cumulative distribution shows that participants above and below these kinks are outliers with regards to the time they spent on the survey. The dominance test and the repeated test were combined with the speed of the survey completion to identify respondents who were quite likely not paying attention to the survey. The sensitivity analysis allowed us to examine the impact, if any, of failing the quality checks on the interpretations of results.

Of the 160 mCRPC patients who responded, 10 failed the quality checks, including 2 (1.3%) straight‐liners, 3 (1.9%) who failed the dominance test and completed the survey in <13 min or >50 min, and 5 (3.1%) who failed the repeated test and completed the survey in <13 min or >50 min. However, no significant changes in preference weights were observed when comparing the preference weights estimated for all surveyed mCRPC patients (*n* = 160) to those who passed the quality check (*n* = 150).

**TABLE B1:** Estimated preference weights

	Preference weight	Standard error	95% CI
Overall survival			
12 months	−0.93	0.16	(−1.24, −0.62)
16 months	−0.26	0.08	(−0.41, −0.11)
22 months	1.20	0.15	(0.91, 1.48)
Months until patients develop a fracture or bone metastasis			
10 months without bone fracture or metastasis	−0.52	0.09	(−0.71, −0.34)
14 months without bone fracture or metastasis	0.05	0.07	(−0.10, 0.19)
20 months without bone fracture or metastasis	0.48	0.11	(0.26, 0.70)
Percentage of patients who need radiation to control bone pain			
20% decrease in bone pain	0.67	0.10	(0.47, 0.87)
30% decrease in bone pain	0.19	0.07	(0.06, 0.33)
50% decrease in bone pain	−0.86	0.11	(−1.07, −0.65)
Worsening of fatigue			
Remain at mild, no worsening	0.41	0.09	(0.24, 0.59)
Worsening from mild to moderate	−0.06	0.07	(−0.20, 0.07)
Worsening from mild to severe	−0.35	0.08	(−0.51, −0.19)
Nausea			
None	0.46	0.12	(0.23, 0.69)
Mild	0.13	0.09	(−0.04, 0.30)
Moderate	0.03	0.09	(−0.14, 0.20)
Severe	−0.62	0.12	(−0.85, −0.40)
Administration			
Daily oral pills	0.17	0.07	(0.02, 0.31)
IV injections (1‐min, every 3 weeks)	−0.08	0.07	(−0.22, 0.06)
IV infusions (1‐hour, every 4 weeks)	−0.09	0.07	(−0.22, 0.05)

Abbreviation: CI, confidence interval; min, minute
